# Comparing heuristic valuation processes between health state valuation from child and adult perspectives

**DOI:** 10.1007/s10198-023-01668-6

**Published:** 2024-02-03

**Authors:** Stefan A. Lipman, Vivian T. Reckers-Droog

**Affiliations:** 1 Erasmus School of Health Policy & Management, Erasmus University Rotterdam, Rotterdam, The Netherlands; 2 Erasmus Centre for Health Economics Research Rotterdam, Erasmus University Rotterdam, Rotterdam, The Netherlands

**Keywords:** EQ-5D-Y, Time trade-off, Child perspective, Heuristics, Discrete choice experiment, Attribute non-attendance, I10

## Abstract

**Objectives:**

Health state valuation assumes that respondents trade off between all aspects of choice tasks and maximize their utility. Yet, respondents may use heuristic valuation processes, i.e., strategies to simplify or avoid the trade-offs that are core to health state valuation. The objective of this study is to explore if heuristic valuation processes are more prevalent for valuation from a 10-year-old child’s perspective compared to the use of an adult perspective.

**Methods:**

We reused existing data in which EQ-5D health states were valued from adult and child perspectives with composite time trade-off (cTTO) and discrete choice experiment (DCE) tasks. Our analyses focused on comparing completion time and responding patterns across both perspectives. We also explored how reflective of a set of heuristic strategies respondents’ choices were in both perspectives.

**Results:**

We found no evidence for systematic differences in completion time across perspectives. Generally, we find different responding patterns in child perspectives, e.g., more speeding, dominance violations, and clustering of utilities at 1.0, 0.8, and 0. Very few heuristic strategies provide a coherent explanation for the observed DCE responses.

**Conclusion:**

Our results provide some, albeit indirect, evidence for differences in heuristic valuation processes between perspectives, although not across all data sources. Potential effects of heuristic valuation processes, such as transfer of responsibility, may be identified through studying responding patterns in cTTO and DCE responses.

**Supplementary Information:**

The online version contains supplementary material available at 10.1007/s10198-023-01668-6.

## Introduction

Many countries recommend the use of EQ-5D instruments for measurement and valuation of quality-adjusted life years (QALYs) in their guidelines for health-economic evaluations. Valuations for EQ-5D instruments are obtained by asking adult respondents to complete composite time trade-off (cTTO) and discrete choice experiment (DCE) tasks [32, 38]. Although both valuation methods elicit adults’ health preferences, the operationalization of these methods is slightly different. In cTTO tasks, respondents are asked to consider time spent in an impaired health state and indicate how many years in full health they consider to be equivalent. In DCE tasks, respondents are asked to indicate which of two health states they believe is best. In both tasks, it is assumed that respondents consider, compare, and trade-off between all aspects of the health states and choose the option (i.e., point of indifference or health state) that maximizes their utility. That is, the linear QALY model [29] applied to cTTO data imposes that the utility of a health state is defined by both its’ duration and the experienced health impairments, and that this utility is maximized. The Random Utility Framework [27] applied to DCE data assumes that the utility of an alternative (i.e., health state) is derived from its characteristics (i.e., dimensions and levels) and that respondents choose the alternative that maximizes their utility (with a margin of random error).

Strong psychological evidence indicates that individuals do not always maximize their utility because of time pressure, limited knowledge or computational capability, and that they resort to impulsive responses under the influence of emotions [18]. Indeed, individuals are boundedly rational and prone to using heuristic decision processes to manage the complexities with which they are confronted in daily life [10, 11]. Rather than maximizing their utility, respondents may, for example, satisfice by choosing the first health state that meets a certain aspiration level (e.g., describing ‘no problems walking about’) in a DCE task. Such respondents use mental shortcuts and engage only in a partial trade-off between the different aspects of the health state(s), or they may even ignore part of the information provided in a choice task [10, 11]. In case their choices are not (solely and fully) based on the information provided, the assumptions underlying the valuation of EQ-5D health states are violated and the obtained utilities biased. Insight into the use of heuristics is therefore important and may allow for correction in the analysis of valuation data [22, 28, 40].

Individuals are more likely to rely on *heuristic decision processes* when time pressure or task complexity is high [41] and when expending effort on decision-making can be avoided [34]. Hence, when respondents are under (in-or external) time pressure, when they consider the valuation tasks to be difficult or emotionally demanding, or when they are not held accountable for their preferences (e.g., because their preferences are elicited in a hypothetical context), respondents may be inclined to use such shortcuts to simplify or even avoid making the trade-offs that are core to the valuation of health states. This is especially relevant considering the valuation of EQ-5D-Y health states. This instrument was developed for measuring health-related quality of life in children aged 8–15 years [6]. The methods recommended for EQ-5D-Y valuation slightly differ from those of the adult EQ-5D instruments [32]. That is, in valuation of EQ-5D-Y-3L, adult respondents are asked to ‘consider their views about a 10-year-old child (henceforth: child perspective) when completing valuation tasks’ (see also: [36]). As such, a layer of complexity is added to the valuation tasks as compared to respondents’ valuation of EQ-5D health states for themselves (henceforth: adult perspective).

Recent quantitative evidence indicates that the change to using a child perspective in valuation of EQ-5D-Y health states yields different valuation outcomes than using an adult perspective in valuation of similar EQ-5D health states [19, 20, 25, 26, 36]. For example, the valuation of EQ-5D health states from a child perspective generally results in (slightly) higher cTTO utilities for similar health states than valuation from an adult perspective [20, 36]. More importantly for the current paper, these studies suggested that the observed differences may (at least, in part) be explained by different valuation *processes* [19, 20, 25, 26, 36]. For example, more respondents refused to trade-off in cTTO tasks when completing the tasks from a child perspective [21, 25, 26]. Furthermore, higher rates of inconsistencies (i.e., violations of dominance) were observed when EQ-5D health states were valued from a child perspective [25].

Recent qualitative evidence may provide substantiation as to why these differences in valuation outcomes and processes occur. In particular, recent studies suggest that the valuation of health states from a child perspective is generally considered more complex by respondents (both cognitively and emotionally) than the valuation of similar health states from adult perspectives [2, 4, 30, 33]. The valuation of EQ-5D health states from a child perspective seemingly leads to (more) inner conflicts and discomfort in respondents. For example, Åström et al. [2] quote participants that found deciding for a 10-year-old child to be ‘horrible’ and feeling ‘grotesque’ when giving up life years for a 10-year-old in cTTO tasks. Summarizing findings across a set of qualitative studies [2, 4, 30, 33], this type of response appeared to apply mostly to cTTO, because participants expressed being uncomfortable with giving up life years for a child. This apparent unwillingness to trading-off between length and quality of life strongly conflicts with participants’ motivation to avoid suffering in children at all costs. Still, participants expressed hesitance with completing cTTO as well as DCE tasks, as they did not feel legitimized to make life (and death) decisions for a child and had difficulty imagining how impaired health would affect another person. Furthermore, Reckers-Droog et al. [33] observed participants ignoring some dimensions in DCE tasks, distancing themselves from the tasks emotionally, and even disengaging completely. Some participants in their study would give up life years in cTTO tasks, but only up to the point where they felt the child for which the task was completed would be able to decide for themselves. For example, in a cTTO task completed with a child perspective, adults are asked how many out of 10 years that a 10-year old child would live in, e.g., extreme pain, they would give for the child to live in full health instead. Some participants in their study would trade off 2 years and declare that further decisions could be transferred to the child from that moment onwards (as the child would be 18 years old and is considered an adult)—which was labeled ‘Transfer of responsibility’. If respondents refused further trade-offs, this would yield utility values of 0.8 in a standard cTTO, task.

Summarizing across these differences in valuation outcomes and processes, Reckers-Droog et al. [33] hypothesized that the valuation of EQ-5D health states from a child perspective could increase the use of processes that make the task easier for respondents as compared to valuation from an adult perspective—and labeled those as *heuristic valuation processes*. Nonetheless, it currently remains unclear whether, and to what extent, such processes influence the outcomes of health state valuation, i.e., the utility of EQ-5D-Y health states. Therefore, the aim of the current study was to explore whether we could identify such processes in valuation data obtained from child and adult perspectives and, if so, to assess whether such processes occurred more frequently in health state valuation from a child perspective and the influence of such processes on the outcomes of health state valuation.

## Methods

Heuristics are typically defined as strategies individuals use that ‘ignore part of the information, with the goal of making decisions more quickly, frugally, and/or accurately than more complex methods.’[11]. In this paper, we define heuristic valuation processes as any process or strategy that may, (un)consciously, be used by respondents to potentially avoid or simplify the trade-offs that are the core of health state valuation tasks. In the following, we describe how we defined and explored the occurrence and influence of various heuristic valuation processes and strategies in valuation data obtained by Kreimeier et al. [20]. We selected (and obtained permission for using) these data for meeting the aim of our study based on the random assignment of respondents to an adult or a child perspective as well as our requirement that these arms were conducted under identical conditions [38], using similar quality-control procedures [31]. The section below provides a summary of the sampling strategy, sample characteristics, study design, and valuation tasks used (and described in further detail) by Kreimeier et al. [20].

### Characteristics of the data obtained by Kreimeier et al. [20]

Kreimeier et al. [20] used convenience sampling strategies to recruit a sample that resembled representativeness in terms of age and sex within Germany, Spain, the Netherlands, and the United Kingdom. Within each country, respondents were randomly assigned to one of four study arms: (1) EQ-5D-3L valued from an adult perspective, (2) EQ-5D-3L valued from a child perspective, (3) EQ-5D-Y-3L valued from an adult perspective and (4) EQ-5D-Y-3L valued from a child perspective. Table 1 presents the sample characteristics by adult and child perspective.

**Table 1:** Sample characteristics

	Perspective	
	Adult (*n* = 399)	Child (*n* = 406)
Age—mean (SD)	42.68 (15.4)	44.19 (16.5)
Sex—*n* (%)		
Male	161 (40.3%)	172 (42.6%)
Female	238 (59.6%)	234 (57.9%)
Education level—*n* (%)		
Low	96 (24.1%)	87 (21.5%)
Medium	147 (36.8%)	156 (38.6%)
High	155 (38.8%)	163 (40.3%)
Parents—*n* (%)	215 (53.9%)	238 (58.9%)
Works with children—*n* (%)	129 (32.3%)	146 (36.1%)
Country—*n* (%)		
Germany	98 (24.5%)	100 (24.8%)
Spain	95 (23.8%)	105 (26%)
The Netherlands	106 (26.6%)	99 (24.5%)
United Kingdom	100 (25.1%)	100 (24.8%
Instrument—*n* (%)		
EQ-5D-3L	205	195
EQ-5D-Y-3L	194	211

Adapted from Kreimeier et al., [20] by merging study arm 1 with 3 and 2 with 4

Within each study arm, respondents first reported demographics and subsequently completed a ranking task in which they were asked to rank the 10 dimension-level descriptors for the respective EQ-5D instrument they were assigned to (i.e., level 2 and 3). Respondents then completed 13 cTTO tasks. A total of 4 cTTO tasks were completed as a warm-up (for health states: life in a wheelchair, 12211, 13222, and 33233), followed by another 9 cTTO tasks (for health states: 11112, 11133, 11312, 13311, 21111, 23232, 32211, 32223, 11113, 11121, 11131, 11211, 12111, 22222, 32313, 33323, and 33333, which were divided into two blocks). Afterwards, respondents completed 27 DCE tasks. These tasks included 9 paired comparisons between two health states: 11332 vs. 22222, 13213 vs. 32331, 11113 vs. 11121, 31212 vs. 12111, 32121 vs. 11211, 31231 vs. 32313, 33323 vs. 21133, 11131 vs. 13222, and 33333 vs. 23333. After each paired comparison task, respondents completed two DCE+death tasks in which the two health states were compared with immediate death (e.g., the paired comparison 11332 vs. 22222 was followed by 11332 vs. immediate death and 22222 vs. immediate death). For details and motivation for selecting these specific health states and the use of DCE + death tasks, see Kreimeier et al. [20].

### Heuristic valuation processes

Based on the qualitative evidence discussed in the Introduction section and on recent work on the use of simplifying heuristics in DCE [40], we identified and explored the use of five heuristic valuation strategies: tallying, take-the-best/lexicographic search, dominant decision-making, attribute non-attendance, and task non-attendance. Table 2 presents an overview of these strategies, their definitions, and potential use in valuation of health states. Note that the set of strategies is not exhaustive as several other heuristics that are potentially influential are not included. We explored heuristic valuation processes and strategies in three sets of analyses focusing successively on the: (i) time-to-complete valuation tasks, (ii) responding patterns, and (iii) heuristic decision strategies. Throughout, we use an exploratory approach, which implies that we will not apply any correction for multiple hypothesis testing.

**Table 2:** Heuristic valuation strategies

#	Heuristic	Definition	Strategy used in EQ-5D health state valuation
1	Tallying^a^	Given the choice between two options, respondents sum across all relevant cues and take the option with the lower (or higher) sum [11]	Respondents pick the health state with the lowest LSS. In cTTO tasks, this would lead to non-trading (utilities of 1) as full health always has a lower LSS than any other EQ-5D health state. In DCE tasks (without duration), this would lead to a choice for the health state with the lowest LSS (e.g., preference for 31113 over 22222) and an indifference for health states with similar LSS
2	Take-the-best/lexicographic search^a^	To infer which of two alternatives is better, respondents go through all cues in order of their importance and decide as soon as one cue discriminates between them. Pick that alternative [7, 12]	To decide which of two health states is best, respondents go through all dimensions in order of their importance and decide as soon as one dimension discriminates between them. Respondents then pick the health state with the best (i.e., lowest) problem level in that dimension. The order of dimensions would likely be idiosyncratic (i.e., different for every person), but can also be determined or defined a priori
3	Dominant decision-making^a^	Given the choice between two options, respondents pick the option that has the best cue value on a single, relevant attribute [40]	Picking whichever health state has the lowest problem level on a single dimension that is considered relevant. For example, given a choice between 23333 and 31111, a respondent satisfying dominant decision-making for the dimension ‘Mobility’ would prefer 23333 (effectively ignoring that state 31111 is a better health state on all other dimensions). All-in trading and non-trading in cTTO may also result from dominant decision-making where the respondent always picks the health profile with better quality of life or the longer duration
4	Attribute non-attendance	Given the choice between two options, respondents completely ignore one (or more) attributes in picking the preferred option [13, 40]	Respondents ignore one dimension in health states and base their preferences on the other four dimensions. For example, when comparing 23123 with state 22222, respondents may (deliberately) ignore the difference in self-care between the health states as they think it is unimportant
5	Task non-attendance	Respondents randomly choose between alternatives (due to lack of information about the validity of cues or lack of engagement) [40]	Respondents that are insufficiently engaged with EQ-5D valuation tasks or are otherwise unwilling (e.g., out of protest) to complete valuation tasks may make (semi-) random choices in valuation tasks

*cTTO* composite time trade-off, *DCE* discrete choice experiment, *LSS* level sum score

^a^Deterministically approached heuristics, i.e., from any given set of alternatives, it is clear a priori which is preferred or if respondents should (rationally) be indifferent. Note that the ‘Take-the-best/lexicographic search’ heuristic is only deterministic in case the search order is known a priori

### Time-to-complete valuation tasks

In general, the time needed by respondents to complete valuation tasks can be seen as a proxy of the level of (perceived) difficulty of the task [41]. In principle, it is intuitive to assume that more complex tasks would require more time to complete. Yet, when a task becomes more complex, respondents may more be inclined to use heuristic valuation processes and, as a result, decrease the time needed for task completion. As such, if the use of heuristic valuation processes is more pronounced in child perspectives due to increased complexity, these tasks would be completed faster. To test this hypothesis, we compare the time needed by respondents for completing cTTO and DCE valuation tasks (in seconds) from a child and adult perspective using Student’s *t* tests. We took into account that respondents generally need more steps to complete a cTTO task that involve more severe health states [38], by also exploring the time needed per step and reporting tests separately for different levels of severity. We further took into account that DCE tasks involve the comparison of two health states and that the time needed to complete a DCE task may depend on the difficulty of that comparison. We used the Level Sum Score (LSS) of health states as a proxy of severity of the health states in time-to-complete estimates for cTTO tasks. For example, the LSS of health state 22222 is 10 and that of health state 33333 is 15. We used the absolute difference in LSS between health states (∆LSS) in time-to-complete estimates for DCE tasks. For example, ∆LSS is 0 for 11332 vs. 22222 and 2 for 13213 vs. 32331, where our assumption is that difficulty decreases with ∆LSS. We arbitrarily set the LSS of immediate death to 16 in time-to-complete estimates for DCE + death tasks to enable comparison.

### Responding patterns

Like in other EQ-5D valuation studies, Kreimeier et al. [20] applied an extensive quality-control procedure and monitored the quality of the choices made by respondents in the cTTO and DCE task along several dimensions [31]. Given that quality-control procedures require that interviewers are trained until a set of predetermined benchmarks for acceptable data quality are reached, data quality is not typically reported on anymore (also not by [20]. Nonetheless, the indicators typically explored as part of standard quality control may yet provide insight into the occurrence of heuristic valuation processes—and are therefore relevant for the aim of this study. For example, some responding patterns that can be considered to contribute to low quality data may result from using heuristics and, as such, may signal that their use is more likely in health state valuation from a child perspective than from an adult perspective. To test this hypothesis, we compare choices made from a child and adult perspective on a set of responding patterns using Chi-squared tests for proportions. We report on these patterns on the level of the respondents and responses (i.e., number of respondents × number of cTTO or DCE tasks).

Responding patterns in cTTO tasks:

- *Clustering of utilities*: Clustering refers to high-frequency occurrence of specific utilities. Typically, clusters around 1, 0.5, 0, and − 0.5, − 1 are considered problematic [1, 31]. Clusters around 1 and − 1 refer to the so-called non-trading and all-in trading (i.e., to floor and ceiling effects) which may cause bias in cTTO responses [16]. High frequencies of responses at 0.5, 0, and − 0.5 may suggest non-engagement (i.e., exiting out of cTTO tasks at early points). We further explore any clustering around utilities of 0.8, as these would signal what Reckers-Droog et al. [33] refer to as 'Transfer of responsibility’.
- *Discriminatory ability:* Ideally, cTTO responses discriminate between health states with different levels of severity. This would, for example, be visible through valuation processes that result in a wide range of unique utilities for different health states within the set of tasks a respondent completes. Following [1, 24–26], we use fewer than 5 out of 9 unique utilities for a set of 9 completed cTTO tasks as a responding pattern of interest. Another way of testing discriminatory ability is by exploring the association between LSS and health state utilities. A typical concern with cTTO responses is the lack of association between LSS and negative utilities [8, 16], which indicates that it is difficult to discriminate between states considered worse-than-dead. Therefore, we compare the discriminatory ability between cTTO tasks completed from child and adult perspectives by calculating Pearson correlation coefficients ($r$) between LSS and utility for (i) all responses, (ii) utilities of non-negative, and (iii) negative utilities. For alternative approaches (and critical notes about the approach applied), see Roudijk et al. [35].
- *Strict dominance violations*: Respondents completed cTTO valuations for health states with different levels of severity, and one would expect that health states that are strictly worse (on all EQ-5D dimensions) receive lower utilities. For example, the utility of state 33,333 should be lower than 31,111 and 33,323. If the opposite is true, this violates dominance. We tested how often such strict violations occurred.

Responding patterns for DCE tasks:

- *Speeding:* Some choice tasks may be completed so fast that they may be a signal of low data quality. For this indicator, we set an arbitrary benchmark of response times below 4 s (i.e., 1/3rd of the median response time across all DCE tasks). Previous work has shown that the use of such arbitrary cut-offs may affect model results and argued for an approach to identify fast responses that uses multiple thresholds [3]. Hence, we also used a 3 and a 5 s benchmark.
- *Flatlining/alternating paths*: Although perhaps unlikely in interviewer-assisted choice settings, respondents may be consistently choosing the left or right health state (i.e., health state A or B) in DCE tasks, or alternatively switching between the two (i.e., left, right, left, right, etc.).
- *Strict dominance violations:* The choice pairs included by Kreimeier et al. [20] offer an opportunity to test for dominance violations, i.e., paired comparison 33323 vs. 33333. Any respondent preferring state 33333 violates dominance (as this health state is worse on all EQ-5D dimensions).
- *Transitivity violations:* Kreimeier et al. [20] operationalized the DCE tasks in three steps by offering respondents paired comparisons between health states, followed by a comparison of each health state with immediate death. This enables us to explore whether respondents’ stated preferences are transitive. That is, for every given set of choices between health states $A, B$, and $D$ (death), we can conclude that if $A\succ B$ and $B\succ D$ than this must mean that $A\succ D$. We test for pattern reversals by reporting the number of responses for which the following holds: $A\succ B, B\succ D, A\prec D$ or $A\prec B, B\prec D, A\succ D$*.* Given that the DCE tasks do not allow indifference, such responses indicate weak transitivity violations.

### Heuristic valuation strategies

A subset of the heuristic strategies summarized in Table 2 could be explored deterministically and statistically in the DCE data obtained by Kreimeier et al. [20].

In the deterministic approach, we define respondents’ expected responses as if they used the heuristic valuation strategies ‘Tallying’, ‘Take-the-best/lexicographic search’, and ‘Dominant decision-making’ deterministically, meaning that they use these strategies consistently and without error. In these cases, we can predict responses on 9 DCE tasks and explore to which extent respondents’ health state valuation from adult and child perspectives are in line with these deterministic predictions (details on these predictions are included as Supplementary Material S1). In case of the strategy ‘Take-the-best/lexicographic search’, we create an idiosyncratic lexicographic search order using the data from the ranking task (see section on data characteristics), in which individuals ranked all dimension-level descriptors. In other words, we assume that respondents search through the dimensions in the order in which they ranked them in a previous task (basing the order on how the 5th level descriptors were ranked). As an alternative potentially viable search order, we also predict how respondents would choose if they applied this heuristic with the order in which the five EQ-5D dimensions appear on the page (henceforth referred to as ‘page order’).

Note that these analyses are only applied to the DCE data, as cTTO data also involve a duration component, and it is unclear how that would influence information search, as well as the prediction for the strategies included. We use the following approach: given a DCE choice task, in which a respondent chooses between state A and B, we determine what each heuristic valuation strategy predicts if all respondents use that particular strategy. This means that respondents either choose state A over B 100% (strategy predicts A is preferred over B), 50% (strategy predicts indifference between A and B), or 0% (strategy predicts B is preferred over A) for a given pair. For each pair of states, we identify if the *actually observed* difference in choice proportions between the perspectives is in the direction predicted by more pronounced use of a heuristic strategy. For example, if all respondents use the ‘Tallying heuristic’, they would always choose 11121 (B) over 11113 (A) as 11121 has fewer problems, yielding a prediction of 100%. As such, if more respondents choose in line with this heuristic in the child perspective, we expect the proportion preferring 11121 over 11113 to be significantly larger in the child perspective than in the adult perspective. On the other hand, the level of problems on the dimension ‘Mobility’ is similar between two, and hence the use of the ‘Dominant decision-making’ heuristic where mobility is used as the dominant dimension (see Table 2) would result in indifference between these health states. Given that the left–right position of health states was randomized by Kreimeier et al. [20], use of this strategy would be expected to result in choice proportions of 50% in this DCE task. If the use of this strategy was indeed more likely in the child perspective, the choice proportions should be closer to 50/50 than in the adult perspective. A final deterministic analysis that we performed is to report for each of the applied strategies how many respondents in each perspective chose 100% consistently (across all 9 choice pairs) in line with the predicted preferences of each heuristic strategy (see Supplementary Material Table S1.1) and compare this proportion across adult and child perspectives.

In the statistical approach, we run a set of multinomial logit models that incorporate components of the strategies ‘, Tallying’, ‘Take-the-best/lexicographic search’, ‘Dominant decision-making’, and ‘Attribute non-attendance’. This means that the logit models explicitly accounted for the assumption that (part of the) information is ignored in the DCE tasks. Regarding the latter two strategies, we explore the effect of restricting the number of EQ-5D dimensions that respondents take into account within a Random Utility Framework. Within this Framework, the utility $U$ of EQ-5D-Y-3L health state *j* typically takes the following form:

1 $${U}_{j}={\beta }_{1}MO{2}_{j}+{\beta }_{2}MO{3}_{j}+{\beta }_{3}SC{2}_{j}+{\beta }_{4}SC{3}_{j}+{\beta }_{5}UA{2}_{j}+{\beta }_{6}UA{3}_{j}+{\beta }_{7}PD{2}_{j}+{\beta }_{8}PD{3}_{j}+{\beta }_{9}AD{2}_{j}+{\beta }_{10}AD{3}_{j}+{\varepsilon }_{j}.$$

In this model (Eq. 1), MO2-AD3 are dummy variables that track the level of problems on each of the EQ-5D dimensions. Given that immediate death has no dimensions, we restrict our analyses to the 9 paired comparison tasks applied by Kreimeier et al. [20]. Note that these comparisons were not intended or designed to efficiently estimate this 10-parameter model, which may explain why this model results in counterintuitive results (see Supplementary Material S2). Given the lack of design efficiency, we simplify the multinomial logit model to the following six-parameter structure:

2 $${U}_{j}={\beta }_{LSS}LS{S}_{j}\left({\beta }_{1}M{O}_{j}+{\beta }_{2}S{C}_{j}+{\beta }_{3}U{A}_{j}+{\beta }_{4}P{D}_{j}+{\beta }_{5}A{D}_{j}\right)+{\varepsilon }_{j}.$$

As such, the relative importance of problems on each EQ-5D dimension are modelled through a single estimate per dimension (${\beta }_{1-5})$, and we include a scaling parameter for severity${\beta }_{LSS}$. This scaling parameter is usually restricted to 1 (and hence, dropped out of the equation), but is relevant for some heuristic models.

The statistical approach to modelling the strategies ‘Tallying’, ‘Dominant decision-making’, and ‘Attribute non-attendance’ is described below:

- *Tallying:* Tallying models imply that health states with the lowest LSS are preferred. To model this strategy, we use one ‘free’ parameter ${\beta }_{LSS}$. All other predictors (${\beta }_{1-5})$ are restricted to 1, indicating that the dimensions each have the exact same weight.
- *Dominant decision-making:* Dominance models imply that decisions are completely and solely based on a single dimension. Hence, we restrict all ${\beta }_{i}$ to 0 except for one specific dimension which then serves as the single determinant of preferences. For example, a mobility-dominant heuristic model implies that ${\beta }_{\mathrm{2,3},\mathrm{4,5}}$ are restricted to 0.
- *Attribute non-attendance:* These models are the inverse of dominance models. Rather than restricting all but one predictor to 0, one single dimension is restricted to 0 to model decisions where this attribute is ignored and hence has no weight in preferences. For example, mobility attribute non-attendance implies that ${\beta }_{1}$ is restricted to 0.

We compare model fit (based on AIC and BIC) for each of these strategies while fixing the parameters for the dimensions that are assumed to be ignored. If respondents’ DCE responses are driven by any of these heuristic decision strategies, we assume that this would result in improved model fit as compared to the standard model [13, 28]. We compare changes in model fit for each strategy. If respondents are indeed more inclined to use any of these strategies in DCE tasks completed from a child perspective than from an adult perspective, improvements in model fit should be more pronounced for the former than the latter perspective. All models were estimated with the Apollo package in R [14].

Note that we only explicitly consider and model heuristics in some of these analyses. For example, when considering responding patterns, it is often unclear which heuristic (if any) is associated with these patterns. For example, consider respondents who have fewer than 5 out of 9 unique utilities in cTTO. This may result from the use of some heuristic valuation process, e.g., non-attendance, but it may be the result from perfectly reasoned preferences. The identification of heuristic valuation processes is further complicated by characteristics of the data collected by Kreimeier et al. [20]. That is, their study was conducted in multiple countries and used two instruments (EQ-5D-3L and EQ-5D-Y, see section on data characteristics) to describe health states. To increase test power, we merged the data obtained from respondents in Germany, Spain, The Netherlands, and the United Kingdom (UK) and discarded differences, such as in wording, between the two EQ-5D. We briefly report on any observed country-specific differences in Supplementary Material S2, and a full by-country transcript of our analysis is available upon reasonable request. Overall, this means that our study can at best provide *indirect* evidence for or against more pronounced use of heuristic valuation processes in child or adult perspectives, which is an issue we reflect on in the Discussion.

## Results

### Time-to-complete valuation tasks

**Fig. 1:**
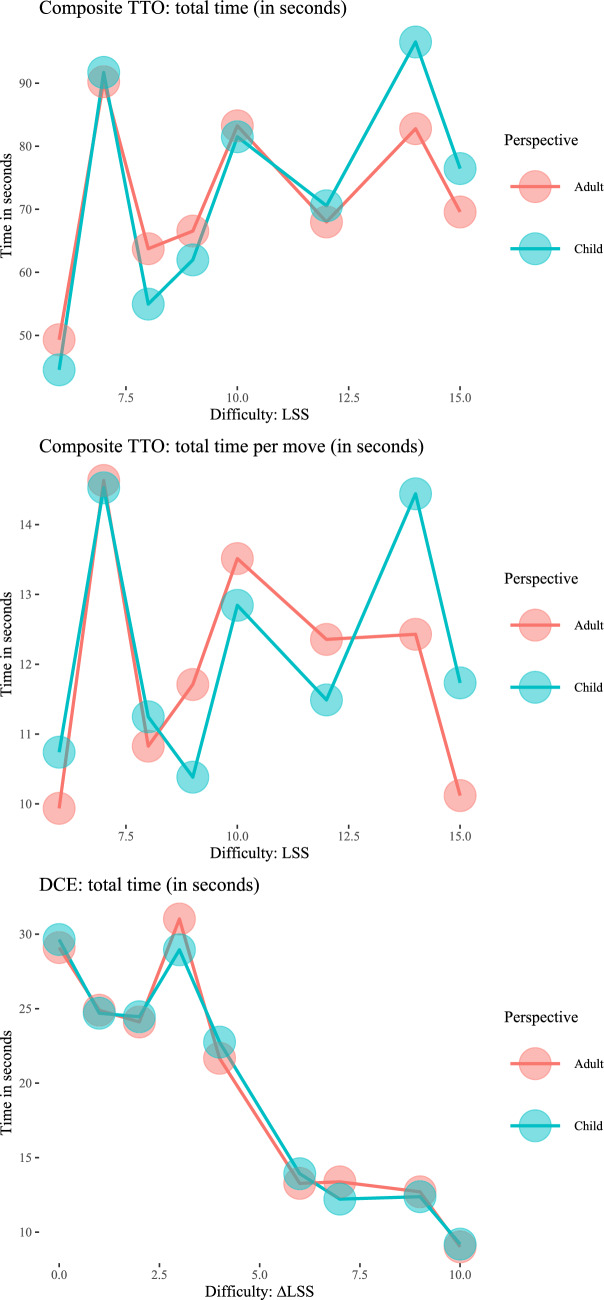
Mean response times by perspective and difficulty (in LSS and ∆LSS)

Figure 1 shows the completion times for the cTTO and DCE tasks (in seconds). The three panels in this figure indicate that the time to complete the valuation tasks may differ little between child and adult perspectives. For cTTO tasks (upper and middle panels), we found no statistically significant difference effect of perspective on completion times across all responses (*t* test, *p* = 0.42). In the Supplementary Material, we show that when repeating this test for health states with different severity, some differences between perspectives can be observed. For some of the mild states (e.g., with an LSS of 6 and 8), cTTO valuation is completed significantly faster in child perspectives than in adult perspectives. In contrast, we found that valuation of severe states with (e.g., LSS = 14 or LSS = 15) systematically takes (marginally) significantly more time to complete from a child perspective than from an adult perspective. For DCE tasks (lower panel), completion times were generally lower for valuation of health states with higher ∆LSS from both perspectives, indicating that the tasks may be considered easier when the absolute difference in LSS is larger between health states.

### Responding patterns

**Fig. 2:**
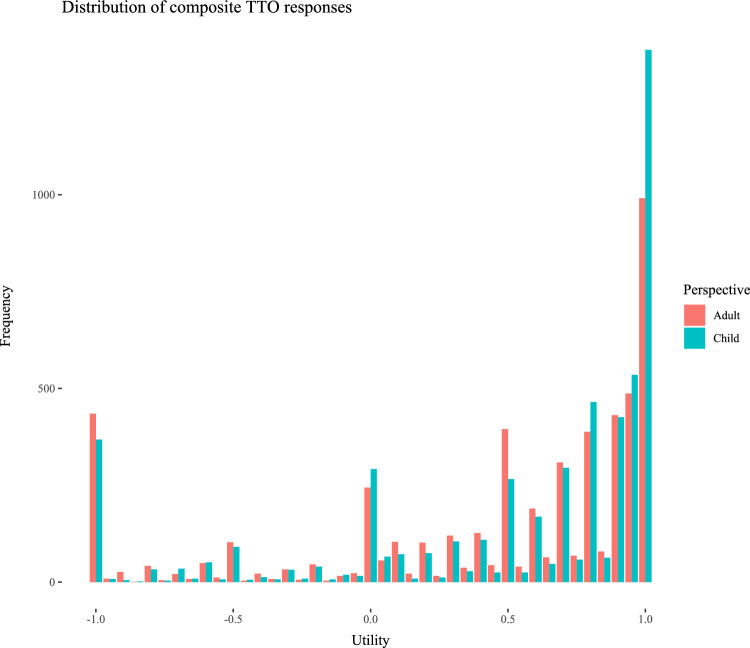
Distribution of cTTO utilities by perspective

Figure 2 shows the distribution of cTTO utilities by perspective and suggests some differences in clustering of utilities between valuation of health states from a child and adult perspective (see also Table 3).

**Table 3:** Responding patterns per type of valuation task, perspective, and level of analysis

	Perspective		Chi-squared
	Adult (*n* = 399)	Child (*n* = 406)	*p* value
cTTO tasks			
Response-level	*N* = 5187	*N* = 5278	
All-in trading (utilities of − 1)	435	368	$p=0.007$
Utilities of − 0.5	103	91	$p=0.36$
Utilities of 0	244	292	$p=0.06$
Utilities of 0.5	395	266	$p<0.001$
Utilities of 0.8	388	464	$p=0.01$
Non-trading (utilities of 1)	991	1374	$p<0.001$
Respondent-level			
Only utilities of − 1, − 0.5, 0, 0.5, 1	23	29	$p=0.55$
Fewer than 5 unique values	114	148	$p=0.002$
Dominance violators	199	226	$p = 0.11$
Correlation LSS and utilities	$r=-0.28,$ $p<0.001$	$r=-0.25,$ $p<0.001$	–
Correlation LSS and non-negative utilities	$r=-0.30,$ $p<0.001$	$r=-0.28,$ $p<0.001$	–
Correlation LSS and negative utilities	$r=-0.26,$ $p<0.001$	$r=-0.23,$ $p<0.001$	–
DCE tasks			
Response-level	N = 10,773	N = 10,962	
Speeding (< 4 s)	884	1028	$p<0.002$
Speeding (< 3 s)	415	478	$p =0.06$
Speeding (< 5 s)	1339	1526	$p =0.001$
Respondent level			
Flatlining/alternating	0	0	–
Dominance violators	3	1	$p=0.60$
Transitivity violators	122	112	$p=0.39$

*cTTO* composite time trade-off, *DCE* discrete choice experiment, *LSS* level sum score

^a^Respondents completed a total of 13 cTTO tasks and 27 DCE tasks

As also shown in Table 3, we find statistically significant differences in the proportion of utilities of − 1, 0, 0.5, 0.8, and 1 for the health states. The direction of differences in clusters is not systematic between child and adult perspectives. For example, we found a higher frequency of clusters at utilities of − 1 (i.e., all-in trading) and 0.5 for valuations from an adult perspective, and a higher frequency of clusters at utilities of 0, 0.8, and 1 (i.e., non-trading) for valuations from a child perspective. Of particular interest may be the cluster at utility of 0.8, which may result from use of the heuristic referred to as ‘Transfer of responsibility’. A relatively higher proportion of utilities of 0.8 could also result from relatively higher utilities attributed to health states valued from a child perspective across all states. However, we found no statistically significant difference in proportion of utilities of 0.7, 0.75, 0.85, and 0.9 (*p* values > 0.17). Supplementary material S2 shows the distribution of cTTO utilities broken down per countries. Some observed differences concern the following: (1) a relatively faster completion of cTTO tasks (in total and per move) when completed from a child perspective in the UK, (2) a relatively higher frequency of clusters at utilities of 0.8 in cTTO tasks completed from a child perspective in Germany and the UK, and (3) a relatively higher frequency of clusters at utilities of -1 (i.e., all-in trading) in cTTO tasks completed from a child perspective in the Netherlands.

Other responding patterns also differed between valuation from adult and child perspectives. When summarizing across respondents, the proportion of dominance violators in cTTO tends to be higher for the child perspective, but not statistically significantly so in the Chi-squared tests (but see Online Supplements S2 for a regression model that reaches significance). The results in Table 3 further show that health state valuation from a child perspective was more likely to yield fewer than 5 unique utilities in cTTO tasks and that speeding in DCE tasks was more likely when health states were valued from a child than adult perspective (regardless of the benchmark used).

### Deterministic models of heuristic valuation strategies

Supplementary Material S1 includes an overview of the expected preferences for deterministic applications of the heuristic valuation strategies for the 9 DCE tasks (i.e., paired comparisons). Table 4 presents the observed choice proportions for each DCE task for both perspectives and shows (marginally) significant differences on 4 out of 9 choice pairs. We indicate for each heuristic strategy whether this difference in choice proportions is in the direction expected when the use of heuristic valuation strategies would be more likely in valuation from a child perspective. Table 4 shows that the only strategy that predicts the significant differences consistently is tallying—in all 4 cases where a significant (*p* < 0.10) difference in choice proportion between perspectives was observed, this was in the direction expected assuming respondents were more likely to rely on tallying in a child perspective. Heuristic strategies using a lexicographic search and decision rule (i.e. take-the-best) showed less consistent results. This strategy would be able to explain 2 of the 4 significant differences observed when using the idiosyncratic search order. A lexicographic search and decision rule based on the order in which the five EQ-5D dimension appear on the page would be able to explain 3 out of the 4 significant differences observed. Dominant decision-making strategies performed reasonably, with 3 out of the 4 significant differences being in the direction expected assuming respondents were more likely to rely on that strategy in a child perspective, with pain/discomfort being the exception. When exploring consistency with heuristic decision strategies at the individual level, our analyses suggest that significantly more respondents chose consistently in line with pain–discomfort dominant decision-making. For dominant decision-making based on anxiety/depression, we find the opposite effect.

**Table 4:** Choice proportions per perspective and indications of increased use of deterministic heuristic decision strategies in DCE tasks completed from a child perspective as compared to an adult perspective

	DCE task									Respondents (*n*) choosing in line with strategy for all 9 tasks		Chi-squared
										Adult (*n* = 399)	Child (*n* = 406)	*p* value
Health state												
A	11332	13213	11113	31212	32121	31231	33323	11131	33333			
B	22222	32331	11121	12111	11211	32313	21133	13222	23333			
Choice proportions (in %)												
Adult perspective (choice A)	10.8	81.5	22.2	2.7	3.0	44.6	45.1	33.3	0.2			
Child perspective (choice A)	10.3	78.2	16.2	2.5	1.5	51.1	26.8	51.1	0.8			
Chi-squared *p* value	0.885	0.275	0.043	1.000	0.248	0.074	< 0.001	< 0.001	0.604			
Deterministic strategy^a^												
Tallying			✔			✔	✔	✔		64	57	0.49
Take-the-best/lexicographic search												
Idiosyncratic order			${\text{X}}$			✔	${\text{X}}$	✔		0	0	–
Page order			${\text{X}}$			✔	✔	✔		1	1	–
Dominant decision-making												
Mobility			${\text{X}}$			✔	✔	✔		33	31	0.84
Self-care			${\text{X}}$			✔	✔	✔		0	0	–
Usual activities			${\text{X}}$			✔	✔	✔		2	0	0.47
Pain/discomfort			${\text{X}}$			${\text{X}}$	${\text{X}}$	${\text{X}}$		15	43	< 0.001
Anxiety/depression			✔			✔	${\text{X}}$	✔		53	29	0.005

^a^Choice tasks in which statistically significant differences in choice proportions indicate increased use of heuristic decision strategy in tasks performed from a child perspective are indicated with ✔ and from an adult perspective with ${\text{X}}$

^b^Total number of DCE tasks (out of the 9 tasks) in which increased use of heuristic decision strategy was observed in tasks completed from a child perspective as compared to tasks completed from an adult perspective

### Statistical models of heuristic valuation strategies

Tables 5 and 6 present the results of the regression models that incorporate components of the strategies ‘Tallying’, ‘Take-the-best/lexicographic search’, ‘Dominant decision-making’, and ‘Attribute non-attendance’ for DCE tasks completed from child and adult perspective, respectively. The results in Table 5 indicate that statistically modelling heuristic strategies using data obtained from DCE tasks completed from a child perspective may not improve model fit.

**Table 5:** Regression coefficients (standard errors in brackets) for statistical heuristic decision strategies in DCE tasks completed from a child perspective (n observations = 3654)

	Parameters^a^							
	${\upbeta }_{{\text{LSS}}}{{\text{LSS}}}_{{\text{j}}}$	${\upbeta }_{1}{{\text{MO}}}_{{\text{j}}}$	${\upbeta }_{2}{{\text{SC}}}_{{\text{j}}}$	${\upbeta }_{3}{{\text{UA}}}_{{\text{j}}}$	${\upbeta }_{4}{{\text{PD}}}_{{\text{j}}}$	${\upbeta }_{5}{{\text{AD}}}_{{\text{j}}}$	AIC	BIC
Standard model	1 (NA)	− 0.862 (0.058)	0.309 (0.062)	− 0.615 (0.05)	− 1.609 (0.095)	− 1.459 (0.071)	3438.13	3469.15
Statistical strategy								
Tallying	− 0.311 (0.015)	1 (NA)	1 (NA)	1 (NA)	1 (NA)	1 (NA)	4602.89	4609.09
Dominant decision-making								
Mobility	1 (NA)	− 0.783 (0.033)	0 (NA)	0 (NA)	0 (NA)	0 (NA)	4354.72	4360.92
Self-care	1 (NA)	0 (NA)	0.315 (0.028)	0 (NA)	0 (NA)	0 (NA)	4941.16	4947.37
Usual activities	1 (NA)	0 (NA)	0 (NA)	− 0.321 (0.031)	0 (NA)	0 (NA)	4960.8	4967.01
Pain/discomfort	1 (NA)	0 (NA)	0 (NA)	0 (NA)	− 0.466 (0.03)	0 (NA)	4801.82	4808.02
Anxiety/depression	1 (NA)	0 (NA)	0 (NA)	0 (NA)	0 (NA)	− 0.035 (0.027)	5065.76	5071.96
Attribute non-attendance								
Mobility	1 (NA)	0 (NA)	− 0.107 (0.046)	− 0.831 (0.049)	− 2.323 (0.086)	− 1.707 (0.068)	3688.98	3713.79
Self-care	1 (NA)	− 0.745 (0.053)	0 (NA)	− 0.526 (0.047)	− 1.905 (0.077)	− 1.569 (0.068)	3461.01	3485.82
Usual activities	1 (NA)	− 0.922 (0.051)	− 0.012 (0.059)	0 (NA)	− 1.63 (0.101)	− 1.497 (0.075)	3603.99	3628.81
Pain/discomfort	1 (NA)	− 1.211 (0.051)	0.988 (0.05)	− 0.474 (0.042)	0 (NA)	− 0.529 (0.041)	3792.6	3817.41
Anxiety/depression	1 (NA)	− 0.966 (0.049)	0.653 (0.053)	− 0.493 (0.042)	− 0.079 (0.048)	0 (NA)	3978.46	4003.28

Strategic heuristic decision strategy modelled indicated in bold

^a^All underlined estimates were fixed at 0 or 1, depending on the strategic heuristic decision strategy modelled (hence the NA standard errors)

**Table 6:** Regression coefficients (standard errors) in brackets for statistical heuristic decision strategies in DCE tasks completed from an adult perspective (n observations = 3591)

	Predictors^a^							
	${\upbeta }_{{\text{LSS}}}{{\text{LSS}}}_{{\text{j}}}$	${\upbeta }_{1}{{\text{MO}}}_{{\text{j}}}$	${\upbeta }_{2}{{\text{SC}}}_{{\text{j}}}$	${\upbeta }_{3}{{\text{UA}}}_{{\text{j}}}$	${\upbeta }_{4}{{\text{PD}}}_{{\text{j}}}$	${\upbeta }_{5}{{\text{AD}}}_{{\text{j}}}$	AIC	BIC
Standard model	1 (NA)	− 0.787 (0.058)	− 0.033 (0.064)	− 0.724 (0.05)	− 1.815 (0.101)	− 1.653 (0.075)	3277.83	3308.76
Statistical strategy								
Tallying	− 0.463 (0.017)	1 (NA)	1 (NA)	1 (NA)	1 (NA)	1 (NA)	4083.86	4090.04
Dominant decision-making								
Mobility	1 (NA)	− 0.843 (0.035)	0 (NA)	0 (NA)	0 (NA)	0 (NA)	4197.34	4203.52
Self-care	1 (NA)	0 (NA)	0.053 (0.028)	0 (NA)	0 (NA)	0 (NA)	4976.56	4982.75
Usual activities	1 (NA)	0 (NA)	0 (NA)	− 0.566 (0.033)	0 (NA)	0 (NA)	4670.22	4676.41
Pain/discomfort	1 (NA)	0 (NA)	0 (NA)	0 (NA)	− 0.264 (0.028)	0 (NA)	4892.15	4898.34
Anxiety/depression	1 (NA)	0 (NA)	0 (NA)	0 (NA)	0 (NA)	− 0.179 (0.027)	4936.29	4942.48
Attribute non-attendance								
Mobility	1 (NA)	0 (NA)	− 0.39 (0.05)	− 0.972 (0.048)	− 2.469 (0.091)	− 1.872 (0.072)	3479.89	3504.63
Self-care^c^	1 (NA)	− 0.799 (0.053)	0 (NA)	− 0.733 (0.047)	− 1.783 (0.079)	− 1.642 (0.072)	*3276.09*	3300.83
Usual activities	1 (NA)	− 0.953 (0.051)	− 0.476 (0.065)	0 (NA)	− 2.028 (0.113)	− 1.824 (0.083)	3502.47	3527.22
Pain/discomfort	1 (NA)	− 1.153 (0.049)	0.726 (0.047)	− 0.604 (0.042)	0 (NA)	− 0.602 (0.043)	3698.92	3723.66
Anxiety/depression	1 (NA)	− 0.899 (0.047)	0.357 (0.05)	− 0.643 (0.043)	− 0.079 (0.048)	0 (NA)	3924.14	3948.88

Strategic heuristic decision strategy modelled indicated in bold

^a^All underlined estimates were fixed at 0 or 1, depending on the strategic heuristic decision strategy modelled (hence the NA standard errors)

The results in Table 6 indicate that this is also the case for modelling data obtained from DCE tasks completed from an adult perspective. However, we find marginally improved fit can be observed when modelling the occurrence of attribute non-attendance for the dimension ‘Self-care’.

## Discussion

The aim of this study was to explore whether we could identify if the use of heuristic valuation processes in health state valuation data obtained from child and adult perspectives and, if so, to assess whether such processes may occur more frequently in health state valuation from a child perspective and the influence of these processes on the outcomes of health state valuation. Based on the available literature on differences in outcomes and processes between completion of cTTO and DCE tasks from these perspectives and the characteristics of the data obtained from Kreimeier et al. [20], we focused our analysis on the time-to-complete valuation tasks, responding patterns, and modelling of deterministic and statistical heuristic decision strategies. Our results provide some evidence for differences in heuristic valuation processes between cTTO and DCE tasks completed from child and adult perspectives; however, we found no systematic evidence suggesting that these processes take place more frequently in valuation tasks completed from a child perspective, as compared to an adult perspective. We further found that heuristic valuation processes may differ between cTTO and DCE valuation tasks.

Overall, we did not find evidence that the time-to-complete cTTO and DCE tasks differed systematically between child and adult perspectives. For mild health states, we find that the time-to-complete cTTO tasks is shorter, which may point in the direction of more pronounced use of heuristics in child perspectives. Yet, in valuations of the most severe health states, we found that the time-to-complete cTTO tasks from a child perspective was relatively longer. An observation that may be of interest is that the time-to-complete cTTO tasks were not monotonically increasing with health state severity (for both perspectives). For example, respondents on average completed cTTO tasks for health state 33333 faster than for health states 33323. This may be a consequence of the left-censoring built into cTTO (utilities are censored at − 1), which is a methodological issue several recent studies have explored [16, 35]. Alternatively, a health state with the worst level descriptors throughout may be quicker to read and interpret. Potentially, this censoring is the result of the use of heuristics, i.e., of respondents avoiding trade-offs by sacrificing all available life years, i.e., yielding all-in-trading responses. In this study, all-in trading occurs more often in adult than child perspectives. Perhaps, this tendency of increased all-in-trading in adult perspectives obscures effects of other heuristic valuation processes, which may be explored by comparing valuation with cTTO methods that allow eliciting a wider range of negative utilities in both adult and child perspectives.

We found some evidence of differences in responding patterns between child and adult perspectives. We found that utilities more frequently clustered at -1 and 0.5 in cTTO tasks completed from an adult perspective, indicating relatively more all-in trading and potentially less engagement with cTTO tasks when completed from an adult perspective [31], respectively. We further found that utilities more frequently clustered at 0, 0.8, and 1 in cTTO tasks completed from a child perspective, indicating a greater tendency to avoid immediate death in cTTO tasks completed from a child perspective (as also observed in previous work [26]), the potential use of the heuristic referred to as ‘Transfer of responsibility’ [33], and a lower willingness to give up any life years [25, 26] in cTTO tasks completed from a child perspective. We found that cTTO tasks completed from a child perspective less frequently resulted in unique health state utilities, indicating that respondents were less able to distinguish between health states for a child than for themselves [25]. We further found evidence for relatively more dominance failures in cTTO tasks and more speeding in DCE tasks completed from a child perspective.

Regarding the clustering of utilities at 0.8 in cTTO tasks completed from a child perspective, we would like to highlight that we observed this cluster *only* at 0.8 and not at utilities around 0.8. As such, this finding may not result from utilities being generally higher for all health states valued from a child perspective, but may indeed result from use of the heuristic ‘transfer of responsibility’ [33]. Interestingly, whereas this transferring of responsibility in child perspectives was identified in qualitative work in the Netherlands, when we compare results of the current study between countries clustering at utilities of 0.8 is not as pronounced in the Netherlands (but rather in Germany and the UK). In our view, the results of this study warrant further exploration of clustering at 0.8, as its’ causes and effects are not entirely clear. Steffel et al. [37] show that such transfer (or delegation) of choice may be especially likely when respondents decide for others, and that respondents may use this strategy over using other simplification strategies. Note that ‘Transfer of responsibility’ does more than simplify decisions, as it also absolves respondents from bearing the potential blame in case decisions for other (i.e., a 10-year-old child in cTTO tasks completed from a child perspective) have negative consequences [33]. This observation may result from respondents strategically absolving themselves from such blame, or from their belief that the child (then adult) will in the future be better equipped at making the decision than they are at this point in time. An alternative explanation for this observation may be that respondents used extrinsic goals as a reference point in composite cTTO tasks [23, 39], because age 18 years may be considered an important milestone for children.

Finally, we found some evidence of differences in the use of heuristic decision strategies between child and adult perspectives. Our modelling approach includes some novel elements, as compared to other studies exploring such strategies in DCE valuation data, which typically focused solely on attribute non-attendance [5, 15, 17]. For example, it includes deterministic predictions for a set of strategies, which allowed us to explore if differences in choice proportions between adult and child perspectives could be explained by these strategies. Furthermore, the DCE data collected by Kreimeier et al. [20] enabled us to define individual-specific ‘Take-the-best/lexicographic’ search orders. Based on the individual rankings of dimension-level descriptors, while assuming respondents search through health states in that same order, we could ‘simulate’ their responses (see Supplementary Material S1). Our results suggest that the heuristic ‘Tallying’ may explain the significant differences between DCE tasks completed from a child and an adult perspective. Although it should be noted that only a small proportion of respondents (~ 15%) showed preferences that in line with this strategy systematically in all DCE tasks and that statistical modelling of this strategy did not improve the fit. We find limited evidence for improved model fit for attribute non-attendance in our regression models (except for modelling attribute non-attendance for the dimension ‘Self-Care’ in data obtained from an adult perspective). This is in contrast to earlier work that generally finds that modelling attribute non-attendance improves model fit [5, 15, 17].

The current study has a set of limitations, which—to the extent that they are data-related—are similar to those described by Kreimeier et al. [20]. Limitations that are specific to the current study concern the following. First, the data collected by Kreimeier et al. [20], for example, included only 9 paired comparisons that we could use for exploring model fit of statistical models incorporating heuristic valuation processes. Further research is warranted to extend (some of) our modelling strategies to DCE valuation data collected using an efficient and tailored design. It is also important to note that all DCE tasks in Kreimeier et al. [20] were completed *after* cTTO tasks. If respondents were already fatigued after completing these tasks, this may have increased the use of heuristic valuation processes irrespective of the perspective that was used (potentially masking differences between perspectives). Second, as we relied on stated preference data, our study did not allow us to identify whether respondents indeed used heuristic valuation processes or whether their preferences simply coincided with such processes. Further research is warranted to determine (with more certainty) the use of such heuristic decision processes. For example, eye-tracking methods [9] could be used to assess whether respondents focus solely or discard (some pieces of) information. Third, our analyses abstract from any potential differences between the EQ-5D-3L and EQ-5D-Y-3L instrument, countries (note that some differences are presented as Supplementary Material S2), as well as any potential effect of respondent characteristics. Potentially, these effects are larger than any potential effect due to heuristic valuation processes, harming our ability to robustly identify the use of heuristic strategies. Finally, throughout our analysis, we have applied a simple approach where we aimed to predict the effects of the use of just a single heuristic valuation process at a time. However, individuals use multiple heuristics interchangeably or even simultaneously. Indeed, boundedly rational individuals are often seen as drawing from an adaptive toolbox, fitting their strategy to the demands of their environment [10, 11].

## Conclusions

Based on the data collected by Kreimeier et al. [20], we find limited evidence for effects of heuristic valuation processes on health state valuation in child perspectives (compared to adult perspectives) on a sample level. However, absence of evidence need not imply evidence of absence. The existing qualitative data strongly suggest that the use of a child perspective increases difficulty of health state valuation, and further research is necessary to examine the consequences of increased difficulty on health state valuation in different perspectives, and EQ-5D-Y value sets. In particular, the clustering observed around cTTO utilities of 0.8 suggests that different decision processes in health state valuation with child perspectives could also affect utilities. In our view, this warrants including this cluster in standard quality-control procedures for studies using child perspectives, as disproportionally large number observations at 0.8 may suggest that respondents are not engaging with the trade-offs underlying valuation methods as expected. We hope that the analyses reported here serve as a starting point for more systematic reporting and exploration of the role of heuristic decision processes in health state valuation, especially when child perspectives are considered. Such future work should also further explore the occurrence of heuristic valuation processes in preference-accompanied outcome measures other than EQ-5D-Y, such as Child Health Utilty 9D or Health Utility Index.

## Supplementary Information

Below is the link to the electronic supplementary material.

Supplementary file1 (DOCX 119 KB)

## Data Availability

Requests for access to this data can be directed to EuroQol: www.euroqol.org. The code used for re-analysis of this data will be shared upon reasonable request.
