# Supplementary material for: Comparing heuristic valuation processes between health state valuation from child and adult perspectives
Source: Eur J Health Econ. 2024 Feb 3;25(8):1345–60. doi: 10.1007/s10198-023-01668-6 (PMC11442527; doi:10.1007/s10198-023-01668-6)
Supplement: Supplementary file 1 — Supplementary file1 (DOCX 119 KB) [file 10198_2023_1668_MOESM1_ESM.docx]

# Supplementary material S1

**Deterministic predictions for heuristic valuation strategies**

Below we outline the predicted preferences for a set of heuristic valuation strategies that are not reliant on trade-offs between dimensions and therefore may yield a deterministic prediction. In order to generate predictions for ‘Take-the-best’ heuristic models, we needed to define a lexicographic search order a priori. We number each search strategy and we provide a brief description of the heuristic model assumed below.

1. Dominant decision-making: mobility – always pick the health state with fewer mobility problems, if mobility problems are the same across both health states one is indifferent.
2. Dominant decision-making: self-care – always pick the health state with fewer self-care problems, if self-care problems are the same across both health states one is indifferent.
3. Dominant decision-making: usual activities – always pick the health state with fewer usual activities problems, if usual activities problems are the same across both health states one is indifferent.
4. Dominant decision-making: pain/discomfort – always pick the health state with less pain/discomfort, if levels of pain/discomfort are the same across both health states one is indifferent.
5. Dominant decision-making: anxiety/depression – always pick the health state with less anxiety/depression, if levels of anxiety/depression are the same across both health states one is indifferent.
6. Take-the-best heuristic: page order: we assume respondents search through dimensions in the order by which they are listed on the page, i.e. mobility, self-care, usual activities, pain/discomfort and anxiety/depression.
7. Tallying – always pick the health state with the lowest LSS, if LSS is the same in both health states one is indifferent.

**Table S1.1** Expected preferences for each of the deterministic heuristic models

|  | **Heuristic decision strategy** | | | | | | |
| --- | --- | --- | --- | --- | --- | --- | --- |
| **Paired comparison (A vs. B)** | **1** | **2** | **3** | **4** | **5** | **6** | **7** |
| 11332 vs. 22222 | A | A | B | B | ~ | A | ~ |
| 13213 vs. 32331 | A | B | A | A | B | A | A |
| 11113 vs. 11121 | ~ | ~ | ~ | A | B | A | B |
| 31212 vs. 12111 | B | A | B | ~ | B | B | B |
| 32121 vs. 11211 | B | B | A | B | ~ | B | B |
| 31231 vs. 32313 | ~ | A | A | B | A | A | A |
| 33323 vs. 21133 | B | B | B | A | ~ | B | B |
| 11131 vs. 13222 | ~ | A | A | B | A | A | A |
| 33333 vs. 23333 | B | ~ | ~ | ~ | ~ | B | B |

**Note:** Whenever the expected preference is A, B or ~, this implies that the expected choice proportions for state A would be 100%, 0% or 50%.

A special case is the idiosyncratic take-the-best heuristic, which leads to an individual-specific prediction (as each individual had their own ranking based on the dimension-level descriptor ranking task). As such, whereas each of the strategies listed above would yield an expected choice proportion of 0%, 50% or 100%, this was not necessarily the case for a lexicographic search order defined at the individual level. Table A2 shows the expected choice proportions (for the whole sample, but also per perspective as the ranking may be different) for each choice pair. The result reported in Table 4 in the main paper merely shows if the child perspective observed choice proportion. is closer to the expected choice proportion than the adult perspective observed proportion.

**Table S1.2. Predicted choice proportions for state A for idiosyncratic take-the-best search order.**

| Paired comparison (A vs. B) | Expected % A  Total sample | Expected % A  Adult perspective | Expected % A  Child perspective |
| --- | --- | --- | --- |
| 11332 vs. 22222 | 40.6% | 39.6% | 41.4% |
| 13213 vs. 32331 | 68.7% | 67.7% | 69.2% |
| 11113 vs. 11121 | 58.5% | 62.4% | 54.2% |
| 31212 vs. 12111 | 9.5% | 11.8% | 7.1% |
| 32121 vs. 11211 | 11.7% | 11.3% | 12.1% |
| 31231 vs. 32313 | 56.7% | 55.1% | 57.9% |
| 33323 vs. 21133 | 47.6% | 48.4% | 46.6% |
| 11131 vs. 13222 | 56.7% | 55.1% | 57.9% |
| 33333 vs. 23333 | 0% | 0% | 0% |

# Supplementary material S2

This Appendix contains results that are referenced but not presented in the main text.

1. Table S2.1 shows the means and standard errors for cTTO time-to-complete both overall, as well as separately for every LSS, as well as reports test statistics.
2. Table S2.2 shows the results for a linear regression model in which the number of dominance violations (per respondent) is taken as dependent, and with the health state block to which respondents were randomly assigned for cTTO and the assigned perspective as independent variables. Block was included in this regression as respondents were assigned to 1 of 2 blocks in the Kreimeier et al (2018) studies, and depending on their block they valued different health states. Obviously, the number of dominance violations will depend on which states are included and how many potentially dominant pairs these yield. In this model, both block and the use of a child perspective are significant predictors, suggesting more dominance violations in valuation from a child perspective. The negative effect of block can be explained by the smaller number of potential dominance violations in the second block compared to the first.
3. Table S2.2 shows the model results for the 10-parameter model outlined in Eq. 1. These results indicate that the sign of parameter estimates goes from positive to negative within dimensions, potentially due to design imbalance.
4. Between country differences in clustering of cTTO utilities (See Figure S2.1)

**Table S2.1**. Mean time-to-complete cTTO tasks by perspective and LSS with standard errors in brackets

|  | **Mean** | **SD** | **Mean** | **SD** | **T-test** |  |
| --- | --- | --- | --- | --- | --- | --- |
| **cTTO utilities** | **Adult perspective** |  | **Child perspective** |  | **T** | **p value** |
| Overall | 71. 20 | 58.13 | 72.20 | 65.25 | -0.77 | 0.441 |
| LSS = 6 | 49.31 | 36.78 | 44.54 | 40.06 | 2.78 | **0.005** |
| LSS = 7 | 90.25 | 69.08 | 91.72 | 71.19 | -0.42 | 0.675 |
| LSS = 8 | 63.75 | 49.47 | 54.96 | 45.69 | 1.86 | **0.063** |
| LSS = 9 | 66.55 | 50.91 | 61.98 | 59.10 | 1.44 | 0.149 |
| LSS = 10 | 83.24 | 69.30 | 81.51 | 71.31 | 0.43 | 0.669 |
| LSS = 12 | 67.99 | 56.22 | 70.56 | 59.52 | -0.77 | 0.440 |
| LSS = 14 | 82.78 | 61.52 | 96.54 | 79.47 | -3.37 | **0.001** |
| LSS = 15 | 69.58 | 52.17 | 76.46 | 64.58 | -1.66 | **0.096** |

**Table S2.2**. Linear regression model results for dominance failures by block and perspective.

| **Dominance violations** | Estimate | **SE** | **T** | **p value** |
| --- | --- | --- | --- | --- |
| Intercept | 1.23 | 0.07 | 16.74 | <0.001 |
| Perspective: Child | 0.19 | 0.09 | 2.20 | 0.029 |
| Block: Block 2 | -0.61 | 0.09 | -7.18 | <0.001 |

**Note:** number of observations: 805, AIC: 2610.8 , BIC: 2629.6

**Table S2.2**. Multinomial logit model coefficients (standard errors in brackets) for adult and child perspective paired comparison data (Eq.1).

|  | **Adult perspective** | **Child perspective** |
| --- | --- | --- |
| $\beta_{1}MO2_{j}$ | -7.89 (1.19) | -9.88 (2.00) |
| $\beta_{2}MO3_{j}$ | -18.98 (18.63) | -20.79 (16.31) |
| $\beta_{3}SC2_{j}$ | 2.46 (0.35) | 3.40 (0.53) |
| $\beta_{4}SC3_{j}$ | -21.19 (18.68) | -23.24 (16.47) |
| $\beta_{5}UA2_{j}$ | 32.14 (37.24) | 35.88 (32.57) |
| $\beta_{6}UA3_{j}$ | 27.91 (37.22) | 30.62 (32.48) |
| $\beta_{7}PD2_{j}$ | 44.48 (55.86) | 49.78 (48.83) |
| $\beta_{8}PD3_{j}$ | 41.12 (55.85) | 46.45 (48.78) |
| $\beta_{9}AD2_{j}$ | -14.36 (18.60) | -15.27 (16.22) |
| $\beta_{10}AD3_{j}$ | 42.84 (55.86) | 48.52 (48.83) |
| AIC | **2814** | **2972** |
| BIC | **2876** | **3034** |

#### Fig. S2.1 Distribution of cTTO utilities by perspective, per country.

**Germany England**

**
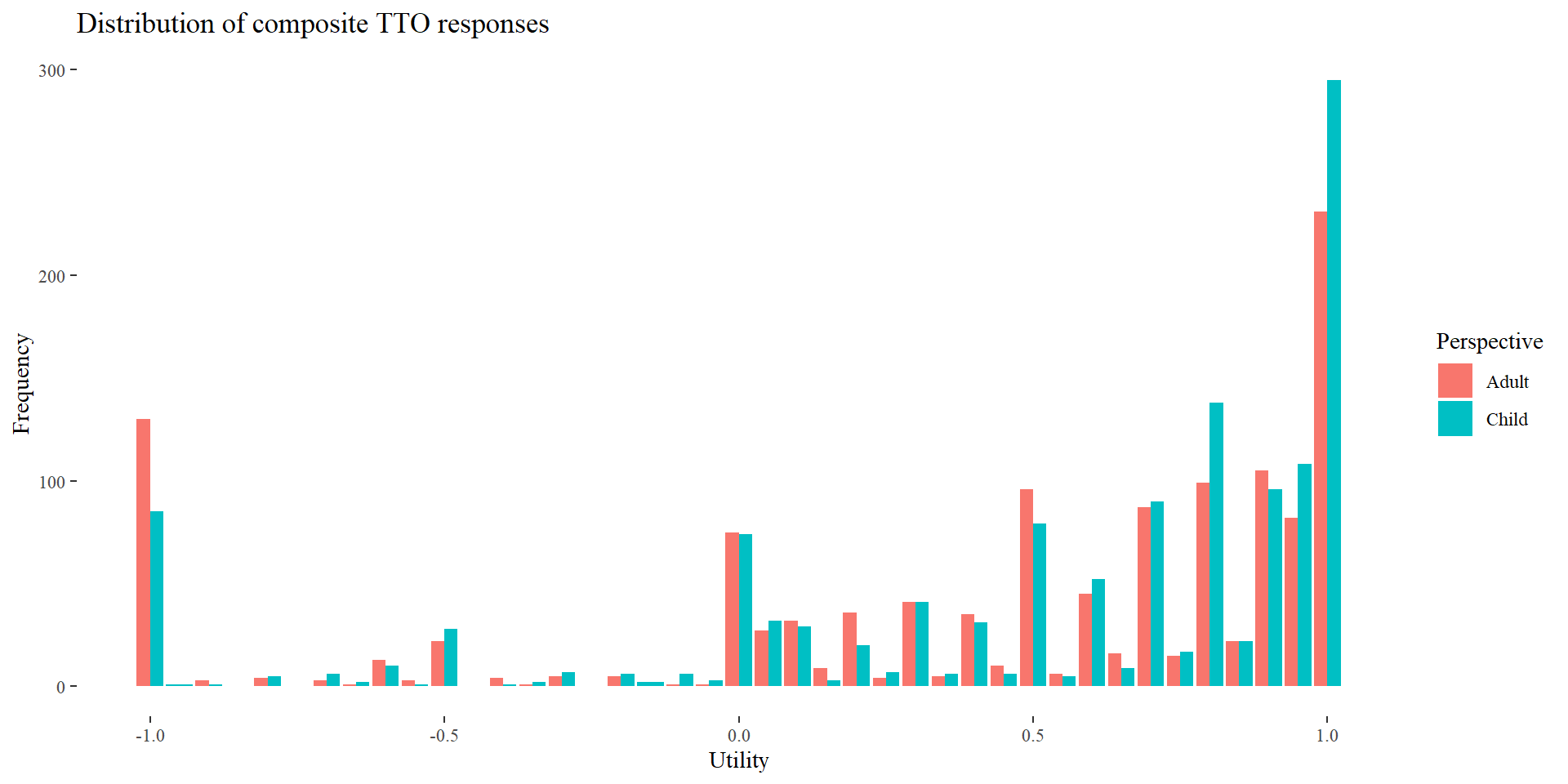

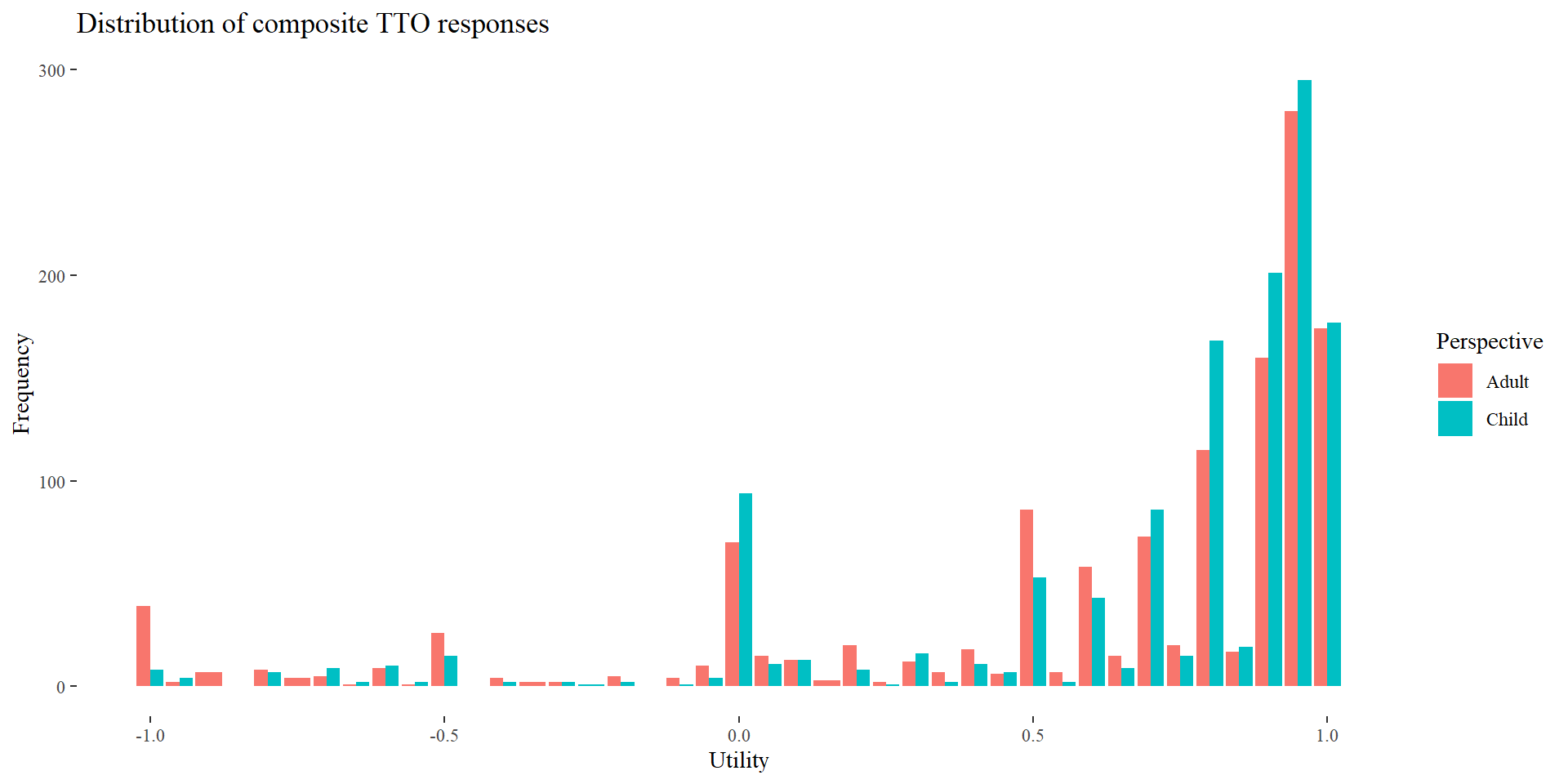
Spain Netherlands
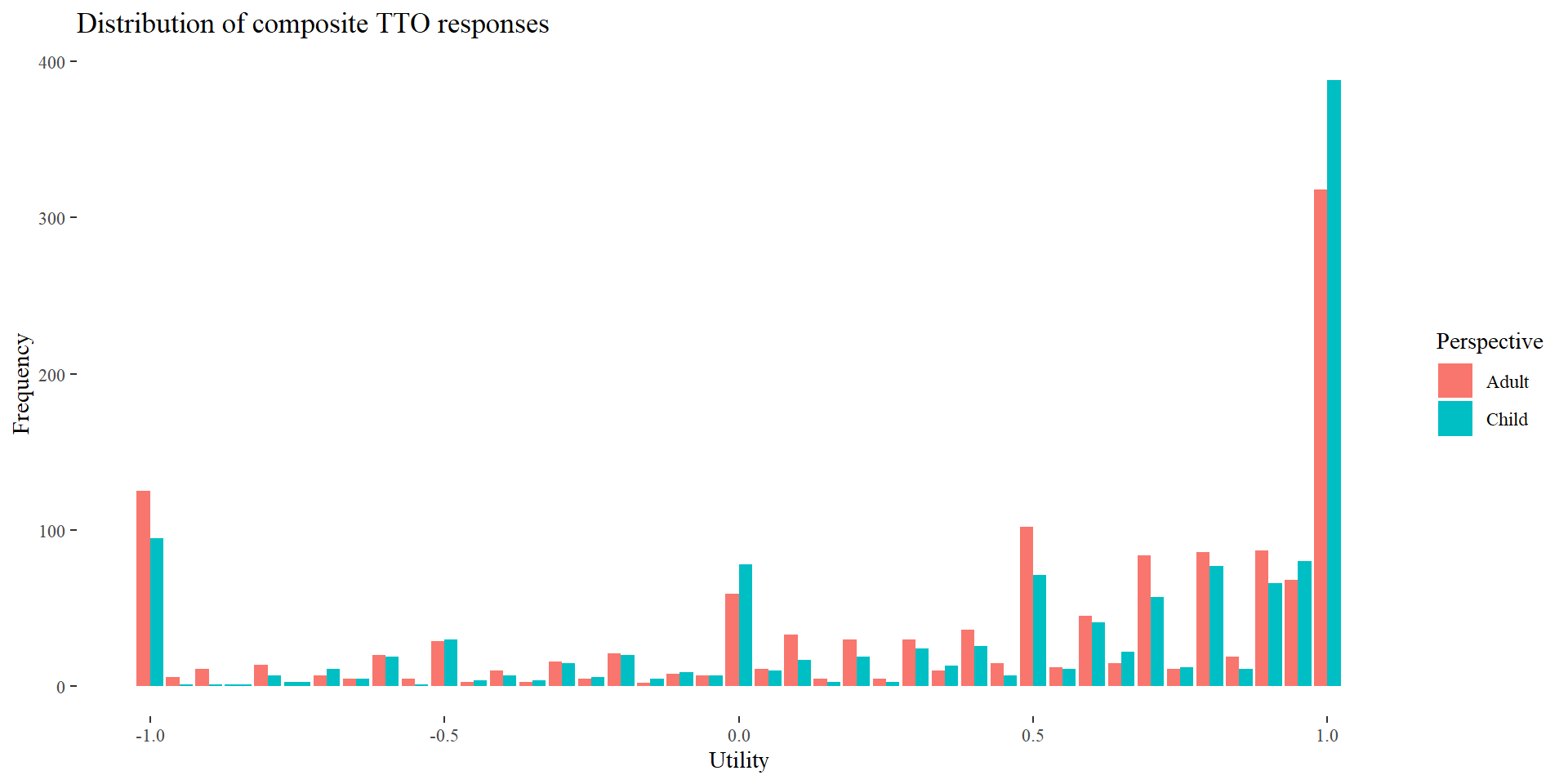

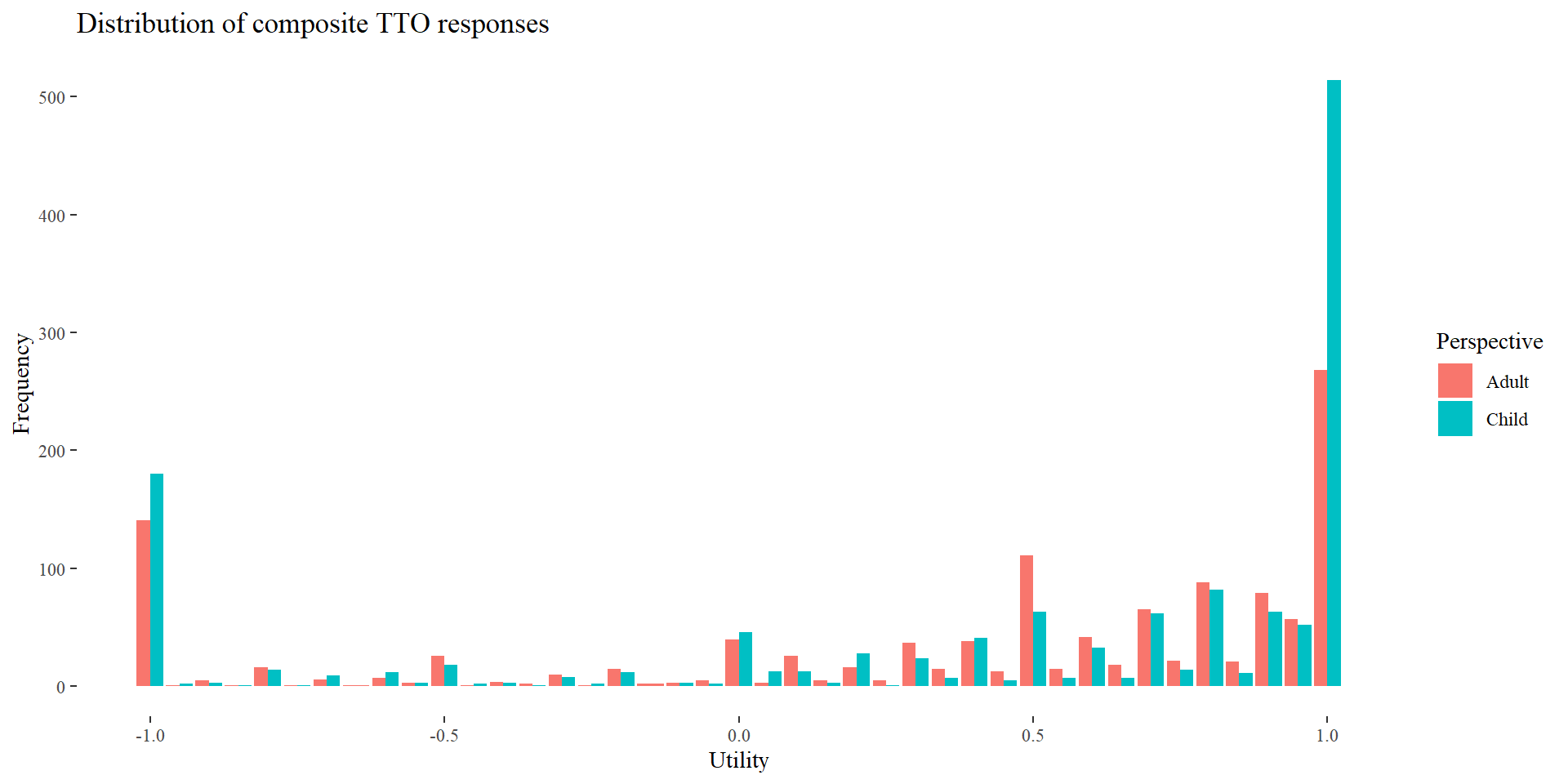
**
